# Axial Compression Experiments and Finite Element Analysis of Basalt Fiber/Epoxy Resin Three-Dimensional Tubular Woven Composites

**DOI:** 10.3390/ma13112584

**Published:** 2020-06-05

**Authors:** Liming Zhu, Huawei Zhang, Jing Guo, Ying Wang, Lihua Lyu

**Affiliations:** School of Textile and Material Engineering, Dalian Polytechnic University, Dalian 116034, China; zlm0501abc@163.com (L.Z.); zhanghuawei199411@163.com (H.Z.); wangying5231@dlpu.edu.cn (Y.W.)

**Keywords:** 3D woven composites with tubular, FEM, axial compression, failure mode

## Abstract

In order to avoid the delamination of traditional tubular composite materials and reduce its woven cost, on an ordinary loom, the three-dimensional (3D) tubular woven fabrics were woven with basalt filament tows, and then the 3D tubular woven composites were prepared with epoxy resin by a hand layup process. The wall thickness of the 3D tubular woven composite was thin, and was only 2 mm thick. Through experiments and finite element method (FEM) simulation, the axial compression properties of the material were analyzed. The results show that the material 2 mm thick has good axial compression performance, the maximum load value of the experiment is 10,578 N, and the maximum load value of the finite element simulation is 11,285 N. The error between the two is 6.68%, indicating that the experiment and simulation have a good consistency. The failure mode of the material is also analyzed through finite element method simulation in the paper, thus revealing the failure stress propagation, local stress concentration, and failure morphology of the material. It provides an effective reference for the design and application of the 3D tubular woven composite.

## 1. Introduction

With the sustained and rapid development of economy and technology, the transportation industry has become increasingly diversified. In addition to traditional roads, railways, waterways and aviation, the pipeline transportation industry is attracting increasing attention. Fiber-reinforced composites are regarded as one of the most important future lightweight materials [1]. The tubular composite material has gradually been valued for its excellent performance. The tubular composite material is a kind of structural original with reasonable stress form, which has the characteristics of light weight, high specific strength, excellent fatigue resistance and good corrosion resistance. Its applications are mainly concentrated in pipeline transportation, fire protection, medical treatment and aerospace [2,3]. The manufacturing process of tubular composite material is generally divided into four categories. They are extrusion molding, yarn wrapping, fabric wrapping, and tubular fabric compounding with resin. Extrusion molding is mainly used in metal processing. Yarn wrapping and fabric wrapping are currently widely used methods. However, due to delamination consolidation, when the material is subjected to alternating external forces, it is easy to cause delamination damage [4]. In the reinforced fabric of the 3D tubular woven composites, the fabric is not only based on the principle of traditional weaving, but also has a vertical yarn system which is introduced in the thickness direction. The upper layer is connected to the lower layer by the vertical yarn, thereby improving fundamentally the phenomenon of cracking between the layers. The woven product is a three-dimensional skeleton, which is directly compounded with the resin. It greatly simplifies the production process and improves the utilization rate of raw materials.

For the weaving of the 3D tubular woven composites, scholars have made continuous explorations. Bai [5] verified the new technology of weaving tubular fabrics on multi-shuttle box looms through the trial weaving of tubular fabric samples. This provides effective guidance for the structural design of the tubular fabric. Zhang [6] summarized the simple method of determining the weave diagram. In the paper, the total warp number of the tubular fabric and the structure relationship between the surface and the inside and the cross-sectional diagram were studied. This provided a theoretical basis for the design and production of tubular fabrics. In terms of the woven fabric with tubular shaped parts, many achievements have also been made. Zheng [7] found an easy way to design and manufacture a woven integrated T-joint tube by the theory of ‘flattening-weaving-unfurling’. A four-layer interlocked hollow woven structure with sandwiched weft wicks in a mini-hole was designed to give the fabric the ability of being extended after part of the wick yarns had dropped out. The final experiment indicates that the three-dimensional T-joint tube can be unfurled smoothly and the method of the design is visually verified to be effective. Huang [8] designed the structure chart and looming draft of T-shaped, I-shaped, triangular and rectangular three-dimensional woven fabrics by using a layered orthogonal method and connection method. The trial weaving on SGA598 semi-automatic sample loom was successful. Bilisik [9] developed multiaxial three-dimensional flat and circular woven preforms and methods for the composite industry. The preform structure consisted of 5 yarn systems. The preliminary experiments verified the feasibility of the weaving methods for manufacturing tubular preforms with different structures. 

Because the performance of the woven tubular composites is mainly reflected in the structural response, damage mode, failure characteristics and energy absorption, it is through the tensile, compression and torsion of the mechanical properties of the material that the structural characteristics of the material can be revealed [10]. In this regard, scholars have conducted a lot of researches. Degrieck [11] presented an experimental investigation on the progressive deformation behavior of unidirectional pultruded composite tubes subjected to an axial impact load. The composites were investigated for three different impact velocities. The crushing peak and mean load characteristics of the composite tubes with different triggering profiles and their progressive failure modes were presented. Rabiee [12] summarized the research results of the past two decades on the crushing process of fiber-reinforced polymer composites tubular structures. The paper provided the considerations of specific parameter definitions in order to design a well-engineered composite structure. This paper created a comprehensive database for researchers, engineers and scientists in the field. Wastiels [13] studied the quasi-static crushing characteristics and the corresponding energy absorption of nine different shapes of composite tubes at small scale. Quasi-static axial crushing tests were conducted to understand the deformation patterns and the corresponding load-deformation characteristics of each composite tube. The effect of dimensions (thickness to diameter ratio) on the specific energy absorption of each composite tube was studied. The results found that the specific energy absorption of special geometrical shapes of the composite tubes is significantly higher than that of the standard and uniform profiles. Sun [14] aimed to investigate the impacts of geometrical parameters on the specific energy absorption of the composite tubes. Finite element models of composite tubes were established. Configurations of these numerical models included circular, square and tapered tubes. Quasi-static axial compression tests were performed to determine failure parameters of glass fiber reinforced polymer used in this paper. Stress-strain curves of the composite tubes were also obtained by tests. Results showed that the specific energy absorption of circular tubes increased with smaller diameter-thickness ratio. Yan [15] investigated the crashworthiness characteristics of natural flax fiber reinforced epoxy composite circular tubes from the point of view of energy absorption. The effects of inner diameter, number of plies and length-to-diameter ratios on mechanical performance of composite materials were researched in detail. by experimental analysis, and the results indicated that the flax fiber-reinforced epoxy composite has a good energy absorption capability. Abdewi [16] researched three geometrical different types of composite tubes subjected to axial and lateral compressive loadings. The results showed that the corrugation geometry in axial crushing significantly affected the loading capability while it does not work in the lateral crushing. Chambe [17] evaluated the ability of various composite structures of circular tubes to dissipate the energy during a compression process. Based on this, several composite tubular structures with different materials and architectures and trigger systems of tubular composites were tested. The results showed that a unidirectional laminate oriented at 0° and stabilized by woven plies strongly met the expectations in terms of energy dissipation. Kim [18] explored the effects of different reinforcing fibers on the crushing behaviors of composite circular tubes. By the analysis of the material properties and the crushing parameters, the results revealed that the fabric carbon/epoxy tubes had the best energy absorption capability whereas the Kevlar tubes showed the worst energy absorption capability. Yan [19] researched the effects of tube thickness, tube inner diameter and the foam filler on the lateral crushing characteristics and energy absorption capacity of composite tubes using a hand lay-up process. The use of polyurethane-foam suppressed the fiber fracturing and eventually enhanced the energy absorption of the tubes during the flattening process. The foam-filled tubes with more fabric plies exhibited better crashworthiness compared to the empty tubes. Additionally, the specific energy of tubes in lateral crushing were significantly lower than those in axial crushing. These works enrich the research on tubular composite material and present the effects of structures and reinforcing fibers on the mechanical properties of tubular composite materials.

It is difficult to fully grasp the performance of the 3D tubular woven composites through observation in macro experiments. In order to further understand the mechanical properties of the 3D tubular woven composites in the microscopic field, theoretical modeling [20] or numerical simulation [21,22] are main methods to conduct research. Compared with the theoretical model which has too many formulas, the advantage of the finite element method is that it can reproduce the failure process of the composite materials through a simple and visual mode failure process. Most importantly, its results are clearly visible. In finite element method (FEM) simulation, Zhu [23] established the geometrical model to analyze the deformation and damage based on the 3D woven composite structure. Compared with data obtained from the experiments and FEM simulation, the results show that the simulated data is in good agreement with the experimental data. This phenomenon proved the validity of the model. Zhou [24] established a meso-structure geometrical model based on the braided architecture to analyze the impact damage and morphology. In the experiment, the load-time histories and deformations of the 3D circular braided composite tubes under transverse impact were obtained. When compared with those in finite element analysis, the finite element analysis results show satisfactory agreements with experimental data and demonstrate the validity of the model. Fan [25] used the progressive failure model of ABAQUS/display materials to simulate the quasi-static compression process of tubular braided composites. The results show that the failure mode, load-displacement curve and calculated energy absorption performance are consistent with the experimental results by the simulation. It proves the effectiveness of simulation. Wang [26] aimed to build theoretical models for the braiding strand trajectories and presented a creative method to establish the parametric geometrical models for the multilayer interlock 3D braided structures with tubular. In the paper, the models of corresponding braiding strand trajectories and braiding structures could be obtained automatically in the simulation environment with the modification of design parameters. The results show that the innovative parametric geometric models of the multilayer interlocking 3D tubular braided structures accurately describe the key characteristics of the preform. Sliseris [27] used numerical simulation and an experimental verification method to research the mechanical properties of hollow and foam-filled flax-fabric-reinforced epoxy tubular composites. In the modelling, a 3D non-linear finite-element model that allowed for the plasticity of materials using an anisotropic hardening model with strain rate dependence and failure was proposed in the simulation process, an explicit dynamic solver was used to address the lateral crashing of the tubes the comparative analysis of results between experiments and finite element method simulation demonstrated the correctness of the finite element method simulation, indicating that this method can be successfully used to design and optimize energy absorbing tubular structures made with flax fabrics to address the problem of overlapped yarns in a 3D four-direction braided tubular composite. Wang [28] presented a modelling approach based on free form deformation theory. The planar and spatial yarn path were analyzed and three geometrical mapping equations are derived from rectangular unit-cell to tubular sub unit-cell. In this way, the model was established. Moreover, the final results validate the feasibility and accuracy of the preform model.

**Figure 1:**
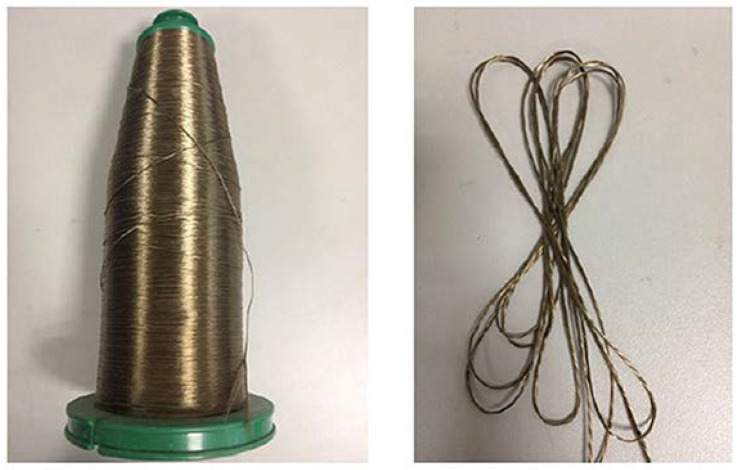
Basalt fiber filament tows.

So far, the research on the 3D tubular composites has mainly focused on the 3D braided tubular composites. There have been few reports on the 3D tubular woven composites. The mechanical properties of the 3D tubular woven composites still need to be further studied. Basalt fiber has excellent chemical resistance, thermal stability, non-toxic, natural and environmental protection [29,30]. Based on ecological considerations, this paper used high-performance basalt filament tows as a raw material to weave the 3D tubular woven fabrics on an ordinary loom, as shown in Figure 1. Then, the 3D tubular woven fabrics were used as reinforcement to prepare composite materials. Finally, the axial compression performance was studied by experiments and FEM.

## 2. Experiment

### 2.1. Materials and Equipment

Eight hundred tex basalt filament tows (Zhejiang Shijin Company, Zhejiang, China) and vinyl epoxy resin V-118 (Wuxi Qianguang Chemical Company, Wuxi, China) were used as the raw materials. A semi-automatic small sample loom (Jiangsu Tongyuan Company, Jiangsu, China) and a universal material testing machine (Jiangsu Tuobo Company, Jiangsu, China) were used for testing.

### 2.2. Design of the 3D Tubular Woven Fabric

**Figure 2:**
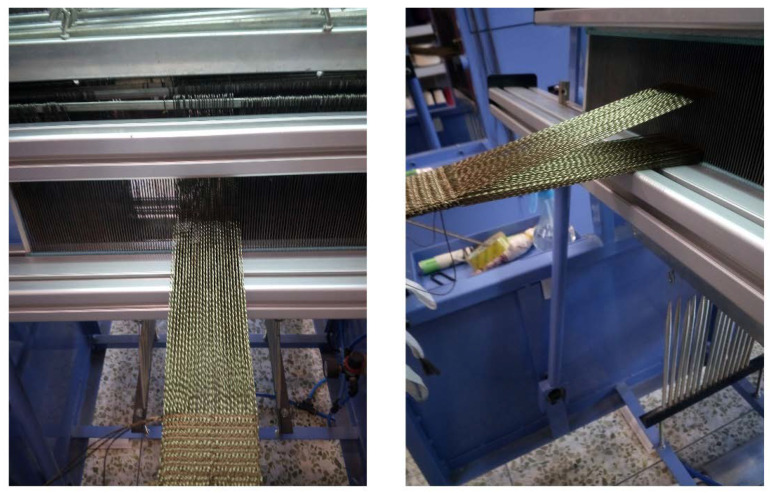
Weaving image of the 3D woven preform on the loom.

The 3D tubular woven fabrics were used as reinforcements for the 3D woven tubular composites. In this paper, the method of warp tube weaving was used. First, some warp yarns were used as vertical yarns. Due to the introduction of a vertical yarn system in the preform structure, the tubular preform was defined as the 3D tubular woven preform. Next, the 3D woven fabrics with tubular of any suitable length were woven by warp tube weaving. Figure 2 showed the weaving image of the 3D tubular woven preform on the loom.

**Figure 3:**
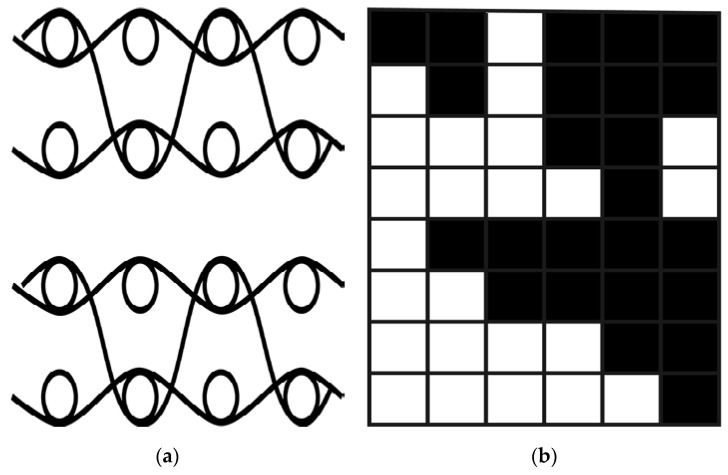
The chain drafts of the 3D tubular woven fabric: (**a**) The warp section drawing; (**b**) The chain drafts.

**Figure 4:**
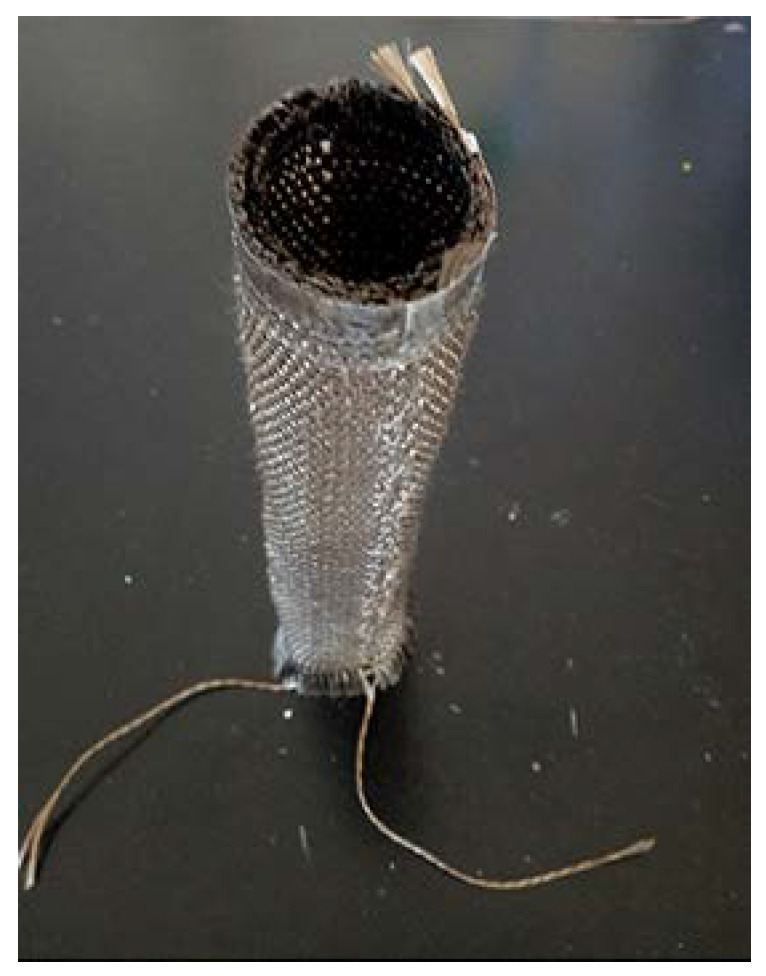
Preform image of the 3D tubular woven preform after its removal from the loom.

The 3D tubular woven fabrics could be simply considered as double pieces of fabric with the same fabric structure. The two pieces of fabrics were connected to form a 3D woven fabric with tubular weft. The meridional section diagram and lamination diagram are shown in Figure 3. The line represented the warp, and the circles represented the weft. According to the number of weft layers in Figure 3a, the sample was defined as a double-layer fabric. During weaving, two weft yarns were inserted alternately to form a fabric. The chain drafts of the 3D tubular woven fabric is shown in Figure 3b. From the warp section drawing of the preform structure, it clearly presented that the vertical yarns cost more yarn length to bind the layers. But all warp yarns including vertical yarns were tight in the actual weaving process and the perform was relatively thin. Hence, the length of vertical yarns was slightly longer than other warp yarns. After uniform beating in the weaving process, good edges of the tubular weaving preform were obtained due to the usage of two continuously alternative weft yarns. Additionally, the yarn looseness of the preform was not significant after its removal from the loom because of the complex yarn connection. Figure 4 shows the 3D tubular woven preform after its removal from the loom.

### 2.3. Preparation of the 3D Tubular Woven Composites

**Figure 5:**
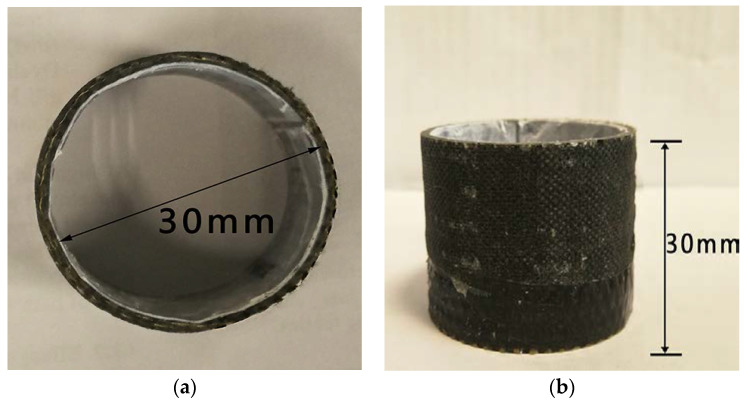
Photograph of the 3D tubular woven composites: (**a**) Diameter image; (**b**) Height image.

The forming process of composites was varied. Tan [31] prepared core material of the tubular materials through the method of a circular ring wax mold, and prepared two-dimensional tubular braid composites by a hand lay-up process. The method was simple and convenient, and the paraffin could be recovered. A paraffin cylinder mandrel of appropriate size was directly poured by paraffin, and the paraffin was reused. Next, because the thickness of the prepared 3D woven fabric with tubular was relatively thin, the hand lay-up process could infiltrate the fabric effectively. Therefore, the hand lay-up process was adopted to form the composite materials. In the pouring experiment, the ratio of chemical reagents (resin: curing agent: accelerant) was 100:5:5. Composite reinforced by the 3D tubular woven preform was defined as the 3D tubular woven composite after curing. Figure 5 revealed the photograph of the 3D tubular woven composites.

## 3. Axial Compression Performance Testing

**Figure 6:**
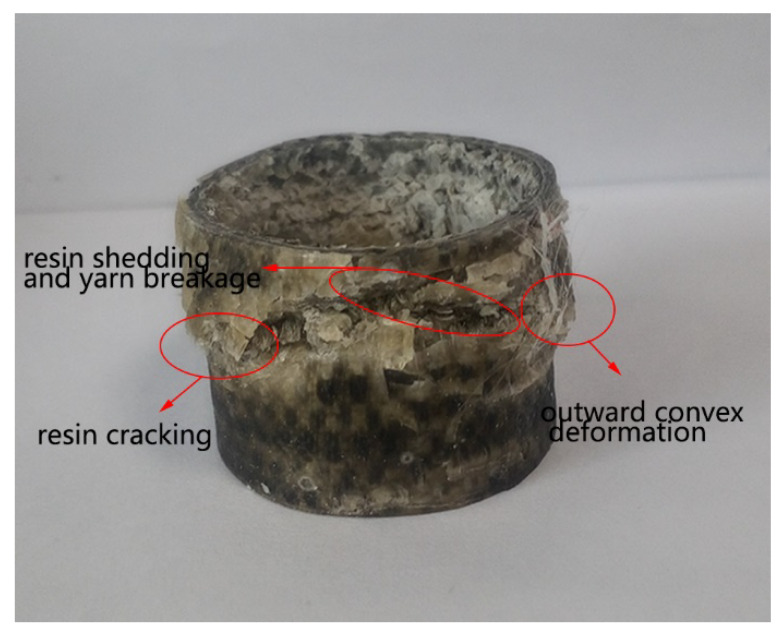
Final failure photograph of the 3D tubular woven composite.

First, referring to GB/T 1446-2005 [32], the 3D woven composites with tubular was prepared with a height of 30 mm, an inner diameter of 30 mm and a wall thickness of 2 mm. Next, the axial compression test was carried out on the material testing machine with the speed set at 10 mm/min at the room temperature. In the experimental process, three samples of the same size were tested under the same experimental conditions. In a compression experiment, usually hundreds to thousands of data points were obtained. With the effect of resin infiltration on the composite structure during the molding process, there were certain differences (i.e., peak load value and corresponding displacement, load value in the third stage, and a certain degree of deviation on curve slope). Finally, the load-displacement curve, the energy-displacement curve and the failure mode were obtained. The final failure photograph of the 3D woven composite with tubular is presented in Figure 6.

## 4. Finite Element Simulation and Analysis

The FEM simulation can analyze the mechanical properties of materials. Through simulating the stress strain and failure deformation of the materials, it could determine the stress concentration area of the materials. Finally, these data could provide a basis to determine the mechanical properties of the materials and guide the structural design of the materials.

### 4.1. Material Model

**Figure 7:**
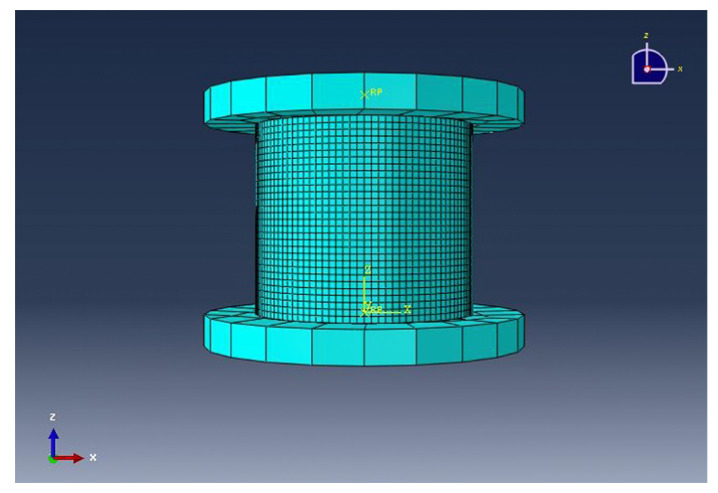
The mesh model of the 3D woven composites with tubular.

According to the size of the material, the three-dimensional deformable solid model of the corresponding size was built in the finite element software. The upper indenter was constrained by the downward displacement, and the bottom plate was correspondingly constrained by the fixed constraint pressure (zero degrees of freedom in all directions). In the meshing of the model, the smaller the meshing of the model is, the more accurate the results. But the computing costs, the time costs, and the performance requirements of computer have also increased. Therefore, in order to simulate the model accurately and protect the computer, the mesh size was set to 1 mm and the total number of hexahedral mesh was 6120. The grid model is shown in Figure 7.

### 4.2. Mechanical Properties of Material

The finite element simulation was input into the finite element software. In the composite material property of the model, the tubular material was set as the elastoplasticity. The corresponding elasticity and plasticity properties were assigned to the composite model. In the plastic properties, plastic stress and strain were input for the FEM simulation, and they were determined according to the mechanical data obtained from the experiment. In addition, engineering constant were input as the elastic parameters. The engineering constant, elastic parameters were determined in the simulation. As for the damage mechanism, the max principal stress law was applied in the simulation of the model. Table 1 shows the mechanical properties of the 3D tubular woven composites.

## 5. Results and Discussion

### 5.1. Load-Displacement Curve

**Figure 8:**
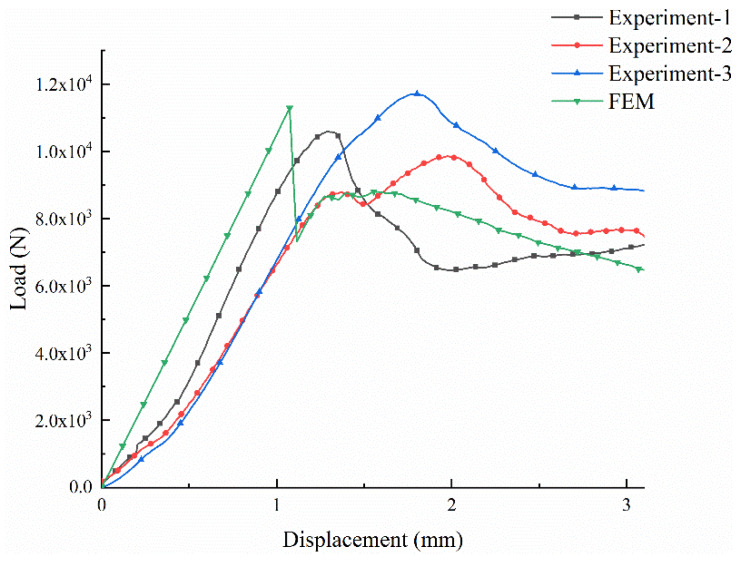
Load-displacement curves of the experiments and finite element method (FEM).

The load-displacement curves of the experiment and FEM are shown in Figure 8. The figure presents the load-displacement curves of the tubular composite material measured three times. The results are represented by the black, red and blue curve, respectively. In addition, the green curve represents the load-displacement curve of the model obtained from the simulation. In the experimental process, three composites of the same size were tested under the same experimental conditions. In a compression experiment, usually hundreds to thousands of data points were obtained. With the effect of resin infiltration on the composite structure during the molding process, there were certain differences (i.e., peak load value and corresponding displacement, load value in the third stage, and a certain degree of deviation on curve slope), as shown in Figure 8. Among the three experimental curves, the experiment-1 curve, the black curve showed a good failure trend and the medium peak load value. Hence, this black curve was selected. In the simulation process, the plastic data input into the model was obtained from the black curve shown in Figure 8.

In Figure 8, the load borne of the 3D woven composites with tubular increases sharply with the increase of the displacement, which indicates that the material is sensitive to displacement. Furthermore, the load-displacement curve can be roughly divided into three stages.

In the first stage, the load-displacement curve increases linearly, because the composite material is fully subjected to stress and exhibits elasticity. As a result of the overall stress, the microcracks are propagated in all structure of the material, and the partial performance is the cracking failure of the resin. In the second stage, the load increases slightly to the peak as the indenter continues to work. At this point, the resin has been damaged a lot and the fiber has started to break, so the curve is no longer linear. In the third stage, after the material reaches the peak load, due to the continued compression of the indenter, a lot of fibers break, and the load that the material can withstand plummets. At this point, the material failure deformation is obvious, thus the material appears to suffer plastic deformation failure. Furthermore, during the subsequent compression process, the load works on the unfractured parts of the composite. Because of the microcrack damage of the whole structure caused by the previous overall compression, the later varying load values are all lower than the peak load. In the early stage, compared with the linear growth of the finite element simulation curve, the experimental curve of the material grows slowly. The reason for this phenomenon may be that the cutting surface is inclined when cutting materials on the cutting machine. When the material is compressed by the universal material testing machine, the pressure plates are initially in partial contact with the surface rather than in full contact. At the beginning, due to the incomplete contact between the pressure plate and the material, the material is not subjected to the overall force. So, the load-displacement curve shows non-linear growth at this moment. With the decrease of the indenter, the load of the material increases gradually. Finally, the load-displacement curve increases linearly when the material is fully stressed.

In general, the 3D tubular woven composites have good axial compression mechanical properties. The maximum load value of the material in the experiment is 10,578 KN, and the maximum load value of the material in the FEM is 11,285 KN. Because the peak load error between experiment and FEM is 6.68%, the FEM curve is in good agreement with the experimental curve. The error analysis table is shown in Table 2. Overall, the FEM results are slightly higher than the experimental data. The reason may be that the model material in the FEM is uniform and consistent. However, in the actual experiment, although the resin mixture is repeatedly coated with the hand lay-up process, there were still some small bubbles inside the material, which affect the mechanical properties of the material. Moreover, in the FEM, the load value drops sharply after material failure, but in the experiment, the load value decreases gradually after material failure. The reason may be that the element integral nodes used in FEM are fewer and the output data points are fewer, but the experimental output data are more. However, in general, the experimental curve is in good agreement with the FEM curve, which indicates the accuracy of the finite element model.

### 5.2. Energy Displacement Curve

**Figure 9:**
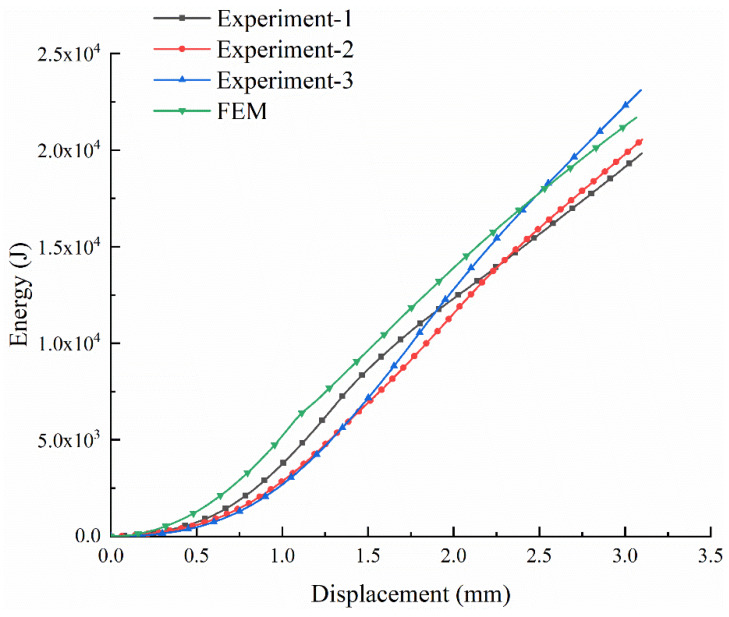
The energy-displacement curves of the experiment and FEM.

Figure 9 shows the energy-displacement curve of the experimental and finite element simulation of the 3D tubular woven composites. The energy-displacement curves of the 3D woven composite with tubular are obtained by integrating the load-displacement curve. The energy absorption of each composite sample is signified by the distinguished curve with different color, corresponding to the load-displacement curve.

It can be clearly seen from Figure 9 that with the increase of the displacement, the energy absorbed by the material under load gradually increases. Furthermore, the difference on energy absorption-displacement curves among these tubular composite materials is negligible. The experiment-1 curve, the black curve, reflects the energy absorption of the selected load-displacement curve with a good failure trend by integrating. At the same time, compared with the experiment-1 curve, the displacement-energy curve of the FEM, the green curve, is slightly higher at each displacement moment. But the two curves show good consistent increasing tendency.

### 5.3. Failure Mode and Mechanism

For the 3D tubular woven composites, it is difficult to judge the stress failure of the materials from the macroscopic perspective only through the experimental method. Compared with the experimental observation, which can only judge the failure mode by observing the final failure state of materials, the FEM can not only obtain the final failure state of materials, but also repeatedly observe the stress propagation, local stress concentration and the failure and deformation of the materials at any time.

**Figure 10:**
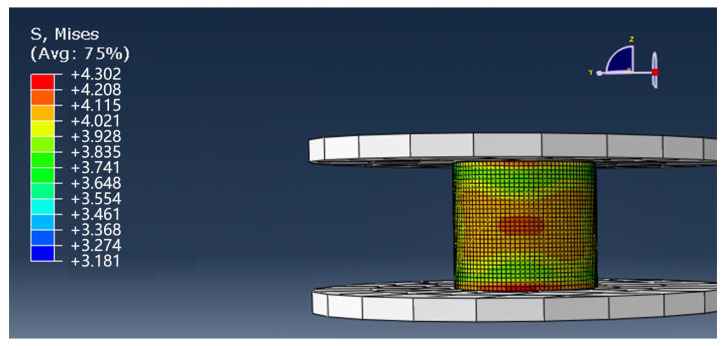
The compression simulation of the 3D tubular woven composites at 0.01 s.

In the FEM compression experiment, the displacement is applied in the opposite direction along the Z axis, so the stress causing the failure of the model is mainly S33 (normal stress along the Z axis). However, due to the continuous downward movement of the indenter during the failure process, S11 (normal stress along the X-axis) and S22 (normal stress along the Y-axis) will also produce corresponding deformation and failure. Therefore, Mises stress was used to express the stress and strain propagation of the entire model. Figure 10 shows the stress nephogram of the 3D woven composites with tubular at 0.01 s.

As can be seen from Figure 10, after the initial compression load is applied to the 3D tubular woven composites, the material behaves in a whole. At this time, the stress concentration region of the material is mainly distributed in the middle region of the tubular composites, and the stress shows good symmetry. At this stage, the overall structural deformation does not occur, but the local resin cracking occurred.

**Figure 11:**
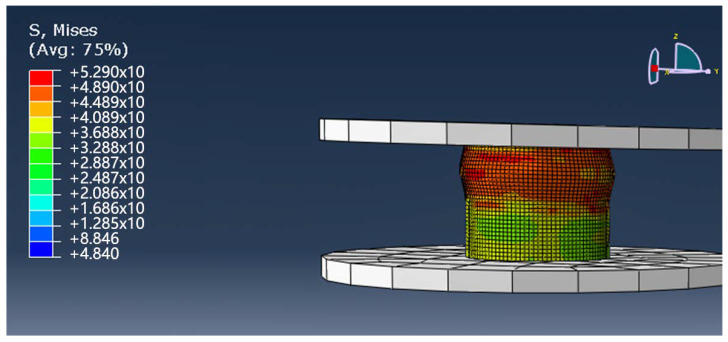
The compression simulation of the 3D tubular woven composites at 0.28 s.

As the upper indenter continues to drop, the axial compression load of the tubular composites continues to increase, and the local stress of the material structure is obviously concentrated on the upper part of the material. As shown in Figure 11, at this point, the material produces outward convex deformation, and the degree of resin damage increases.

**Figure 12:**
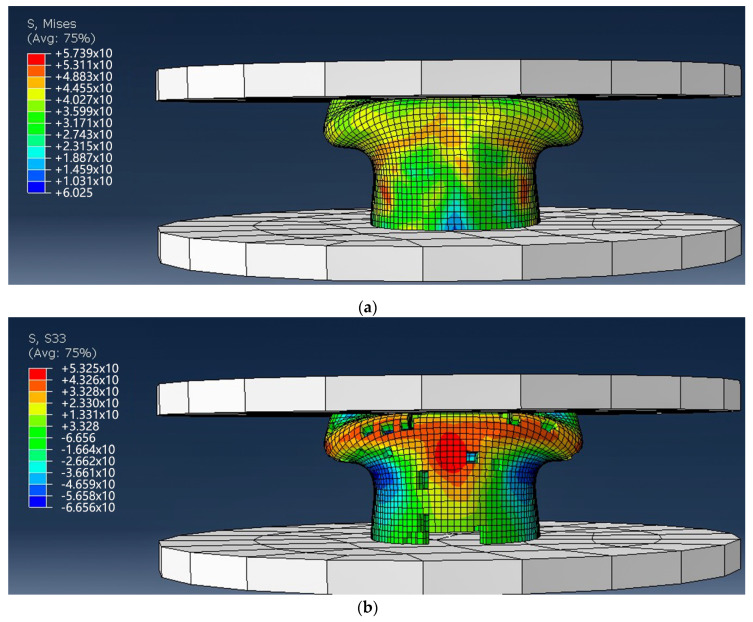
The final failure photograph of the 3D tubular woven composites. (**a**) The final failure photograph of the 3D tubular woven composites under Mises; (**b**) The final failure photograph of the 3D tubular woven composites under S33 direction.

As the upper indenter continues to drop, the 3D tubular woven composite reaches its peak load. At this time, the material produces irregular deformation and failure, which is manifested by obviously local folding deformation on the material surface. As shown in Figure 12a, through further processing the model of deletion failure elements is obtained. At this time, it is found that the failure model of the S33 direction stress-strain nephogram is the most significant. As shown in Figure 12b, where the material crumbles and deforms, a lot of elements are removed. This is because the 3D tubular woven composite is an anisotropic composite, obtained by compounding anisotropic yarn and isotropic resin, unlike the isotropic metallic material. Based on this characteristic, the model in the end generates asymmetrical failure deformation after the axial compression simulation.

After the experiment, by observing the broken 3D tubular woven composites in the upper fold area of the composites, the resin cracks and falls off in a large area, and the yarn fracture is obvious. As presented in the Figure 6, the compression failure occurs at the upper middle region. It shows outward convex deformation which is highlighted in the image and local fold deformation in the yarn breakage region. In the compression process, resin cracking and the shedding phenomenon are most significant. After the compression process, yarn breakage can be clearly observed through the resin shedding area along the failure deformation region. Additionally, after the material is removed from the universal material testing machine, the composite slowly has a certain degree of shape recovery ability. In Figure 12b, the composite model clearly manifests outward convex deformation and local fold deformation. Besides, the removed failure elements along the deformation region are obvious. Combined with the failure deformation and resultant values obtained from the simulation, good consistency is presented between the model and the composite. Hence, it is concluded that the finite element simulation results are in good agreement with the experimental results.

## 6. Conclusions

Through rational design in ordinary looms, green basalt filament tows were used to successfully weave the 3D tubular woven fabrics. The 3D tubular woven fabrics acted as reinforcement, and at the same time epoxy vinyl resin was used as a basal body. The 3D tubular woven composites were prepared by hand lay-up process and the axial compression properties of the 3D tubular woven composites were tested. The experimental results show that the peak load of the two-layer 3D tubular woven composite reaches 10,578 KN. It is concluded that the composites have good mechanical properties. At the same time, the finite element simulation method was used to establish the model of the same size as the experimental material, and then the axial compression simulation was carried out. The results show that the peak load is 11,285 KN and the error is 6.68%. In general, the finite element simulation curve is consistent with the experimental curve, which can prove the accuracy of the finite element simulation model. Based on the correct model failure course, the failure course and the key failure states of the 3D tubular woven composites under axial compression were analyzed. The results show that under axial compression, the changes of the 3D tubular woven composites at each stage are consistent with the experimental curves. Moreover, based on the stress propagation, stress concentration and failure morphology of the material in the failure process, the design and application of materials can be targeted.

## Figures and Tables

**Table 1:** Mechanical properties of the 3D tubular woven composites.

E11/Gpa	E22/Gpa	E33/Gpa	ν12	ν23	ν13	G12/Gpa	G13/Gpa	G23/Gpa
11.6	1.5	1.5	0.25	0.25	0.18	9.5	9.5	6

Note: E11 is the longitudinal elastic modulus of the composite material; E22 and E33 are the transverse elastic modulus of the material; ν12 and ν13 are longitudinal Poisson’s ratio of composite materials; ν23 is the transverse Poisson’s ratio of the material; G12 and G13 are longitudinal shear modulus of composites; G23 is the transverse shear modulus of the material.

**Table 2:** The peak load error of the experiment and FEM.

Test Velocity/(mm × min^−1^)	FEM/KN	Experiment/KN	Error/%
10	11,285	10,578	6.68
